# Evaluation of Microcirculation at the Optic Nerve Head in Eyes With Keratoconus

**DOI:** 10.1167/iovs.67.4.25

**Published:** 2026-04-13

**Authors:** Sera Ichinohasama, Hiroyuki Namba, Tomoaki Shiba, Tatsuhiko Kobayashi, Nene Okamoto, Tomoyuki Kurihara, Marie Ikeda, Yuichi Hori, Tomohiko Usui

**Affiliations:** 1 Department of Ophthalmology, International University of Health and Welfare Narita Hospital, Narita, Chiba, Japan; 2 Department of Ophthalmology, Toho University Omori Medical Center, Ota, Tokyo, Japan

**Keywords:** keratoconus, optic nerve head, microcirculation, ocular blood flow, laser speckle flowgraphy

## Abstract

**Purpose:**

To evaluate microcirculation at the optic nerve head (ONH) in eyes with keratoconus.

**Methods:**

Laser speckle flowgraphy (LSFG) was performed in patients with keratoconus at the International University of Health and Welfare Narita Hospital. Age- and sex-matched control and high myopia groups were selected from screening participants at the Tokyo Kamata Medical Center. LSFG parameters assessed at the ONH included the mean blur rate (MBR) of vascular area (MV), tissue area (MT), and area ratio of bloodstream (ARBS). Values were compared among the three groups, and MV and MT were also analyzed by quadrants.

**Results:**

Thirty-one keratoconus eyes, 31 control eyes, and 26 high myopia eyes were analyzed. For overall ONH, MV was lower in keratoconus than in controls (*P* = 0.040), and MT was lower than in controls and high myopia (both *P* = 0.014). In quadrant analysis, MV was lower in the superior quadrant than in controls (*P* = 0.008). MT was lower in the temporal quadrant than in controls (*P* = 0.022) and high myopia (*P* < 0.001), and in the nasal quadrant than in controls (*P* < 0.001) and high myopia (*P* = 0.031). ARBS was lower in keratoconus than in controls (*P* = 0.001) and high myopia (*P* = 0.036).

**Conclusions:**

LSFG showed keratoconus eyes had significantly reduced MBR and ARBS at the ONH compared with normal and highly myopic eyes. These findings suggest that keratoconus may involve alterations in ONH circulation, highlighting the need to monitor optic nerve function in clinical practice.

Keratoconus is a progressive bilateral disorder characterized by corneal thinning and anterior protrusion, leading to irregular astigmatism and reduced visual acuity. It has traditionally been regarded as a relatively rare condition. However, recent epidemiological studies have revealed that keratoconus is more common than previously thought. Hashemi et al.^1^ reported a global prevalence of 1.38 per 1000 individuals (95% confidence interval [CI], 1.14–1.62) in a meta-analysis including over 7 million participants across 29 countries. A recent Japanese population-based study found that 0.85% of individuals aged over 35 years were diagnosed with keratoconus, and an additional 1.46% were classified as keratoconus suspects.^2^ These findings indicate that keratoconus is not uncommon in clinical practice.

Although keratoconus primarily affects the cornea, accumulating evidence suggests that its pathophysiology may extend beyond the anterior segment. Biochemical studies have demonstrated reduced expression of collagens in the corneal stroma as well as increased activity of matrix metalloproteinases (MMPs) and inflammatory cytokines.^3^^–^^5^ Because collagen is a fundamental structural component not only of the cornea but also of the sclera and lamina cribrosa, keratoconus may involve extracellular matrix abnormalities affecting the entire eye.^6^

Consistent with this hypothesis, several posterior segment changes have been reported in keratoconus.^3^^–^^8^ For instance, the choroid is significantly thicker in keratoconus,^3^^–^^5^ whereas the sclera and lamina cribrosa are significantly thinner.^6^ Furthermore, OCTA studies have found reduced peripapillary vessel density in eyes with keratoconus compared with normal eyes.^7^^,^^8^ These findings collectively raise the possibility that keratoconus is associated with structural and circulatory changes at the optic nerve head (ONH). However, to our knowledge, no studies have directly examined ONH blood flow in keratoconus. Recently, laser speckle flowgraphy (LSFG) has provided a noninvasive method for quantifying ocular blood flow, and the mean blur rate (MBR) has been validated as a surrogate for ONH microcirculation.^9^ In this study, we applied LSFG to evaluate ONH microcirculation in patients with keratoconus and compared the results with those of normal and highly myopic eyes. A high myopia group without other ocular diseases was also included to distinguish keratoconus-related changes from those attributable to high myopia, as advanced keratoconus may present with high myopia.

## Methods

### Participants

We retrospectively analyzed patients with keratoconus who underwent LSFG at the International University of Health and Welfare, Narita Hospital, between April 2020 and February 2025. Age- and sex-matched controls and the high myopia group were selected from individuals who underwent ophthalmic screening at Tokyo Kamata Medical Center between December 2016 and December 2018. In the control and high myopia groups, spherical equivalent and cylindrical power were measured using an autorefractometer (Tonoref 2; Nidek Co., Aichi, Japan). In the keratoconus group, these values represent the refractive correction used to achieve best-corrected visual acuity. Best-corrected visual acuity was converted to logarithm of the minimum angle of resolution (logMAR) units for analysis. The control group included eyes with a spherical equivalent between −3.0 D and 3.0 D, and the high myopia group included eyes with a spherical equivalent of ≥−6.0 D. Only one eye per participant was analyzed. Eyes with coexisting ocular diseases, such as glaucoma or diabetic retinopathy, were excluded from the study across all groups.

This study adhered to the tenets of the Declaration of Helsinki and was approved by the Ethical Review Committee of the International University of Health and Welfare (approval number: 23-Nr-024). Informed consent was obtained through an opt-out option on the website, and those who declined participation were excluded. Personal information was separated from ophthalmic data using linkable anonymization.

### LSFG

In this study, LSFG (LSFG-NAVI; Softcare Ltd., Iizuka, Japan) was used to assess blood flow in the ONH. The device uses an 830 nm near-infrared laser to illuminate tissue, and scattered light reflected by erythrocytes forms a speckle pattern. Temporal changes in this pattern were analyzed to calculate blood flow velocity, producing a color-coded map of flow distribution. The MBR represents the average rate of speckle blurring within a cardiac cycle; a higher MBR indicates faster erythrocyte movement. The overall MBR of the vascular area was defined as MV, and that of the tissue area as MT.

**Figure 1.**
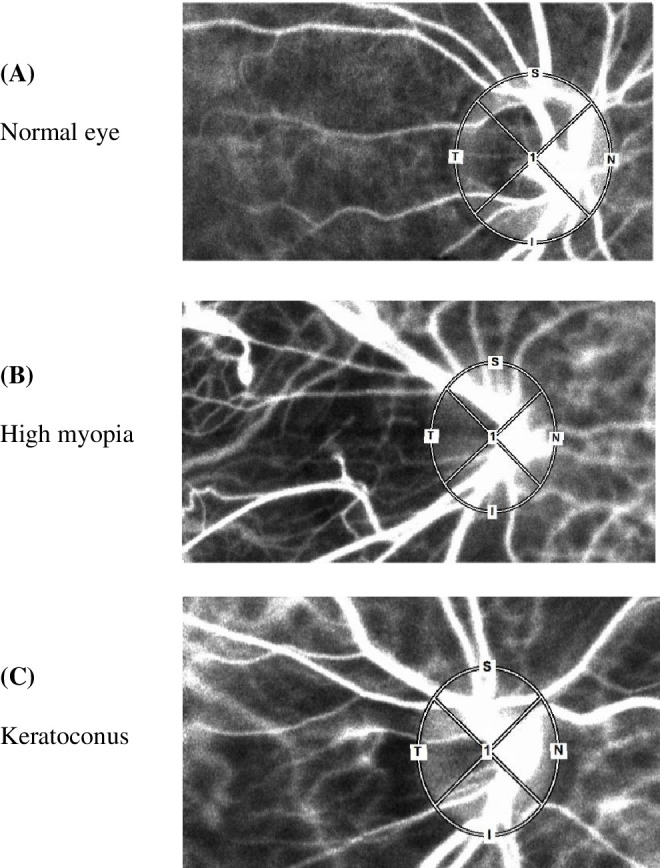
Representative examples of ONH delineation for LSFG analysis in normal **(A)**, high myopia **(B)**, and keratoconus eyes **(C)**.

Additional parameters included the area ratio of the bloodstream (ARBS), representing the proportion of retinal vascular area within the ONH. MV, MT, and ARBS were compared among the control, high myopia, and keratoconus groups for the entire ONH. MV and MT were also analyzed by quadrants (superior, temporal, inferior, and nasal; Fig. 1). All measurements were performed without contact lenses. Because reliable LSFG measurements are difficult to obtain in advanced keratoconus, this study mainly included eyes with mild to moderate keratoconus.

### Pulse Waveform Parameters

As a secondary analysis, we assessed pulse waveform parameters derived from MV, and MT. Parameters included skew, blowout score, blowout time, rising rate, falling rate, flow acceleration index, acceleration time index, and resistivity index. Detailed formulas for these LSFG-derived parameters are available in the reference.^10^

Pulse waveform parameters derived from MBR reflect both ocular circulation and systemic vascular status. For example, skew and blowout times are associated with arteriosclerosis and correlate strongly with the Cardio-Ankle Vascular Index,^11^ whereas acceleration time index is linked to left ventricular function.^12^ Because these indexes may aid in understanding pathophysiology, we compared pulse waveform parameters from MV and MT among the three groups.

### Statistical Analysis

To compare the characteristics among the three groups, Kruskal–Wallis tests with post hoc Dunn's tests were applied. The sex ratio was analyzed using a χ^2^ test. ANOVA was used only for heart rate and mean blood pressure because these variables were normally distributed. MV, MT, and ARBS were analyzed using ANOVA with the Tukey–Kramer post hoc test. Pulse waveform parameters, which were not normally distributed, were analyzed using the Kruskal–Wallis test. Post hoc power analyses were conducted to describe the accuracy of the between-group comparisons using G*Power version 3.1.9.7 (Heinrich-Heine-Universität Düsseldorf, Düsseldorf, Germany).^13^ A power (1 − β) > 0.80 was considered high statistical power.^14^

## Results

### Patient Background

The study included 31 eyes in the keratoconus group, 31 in the control group, and 26 in the high myopia group. The mean age in the keratoconus group was 38.3 years (Table 1). No significant differences were found among groups in age, sex, heart rate, blood pressure, and intraocular pressure. Although the Kruskal–Wallis test revealed significance in visual acuity, the post hoc analysis did not. Significant differences were observed in spherical equivalent and cylindrical power (*P* < 0.001 for both). For spherical equivalent, values were −0.87 ± 0.88 D in controls, −7.16 ± 1.20 D in high myopia, and −5.67 ± 4.39 D in keratoconus, with significant differences observed between the control and high myopia groups and between the control and keratoconus groups, but not between the high myopia and keratoconus groups (*P* = 0.112) with effect size *f* = 0.942, power 1.00. For cylindrical power, values were −0.46 ± 0.45 D in controls, −0.77 ± 0.52 D in high myopia, and −2.70 ± 2.36 D in keratoconus, with significant differences between controls and keratoconus and between high myopia and keratoconus (*P* < 0.001 and *P* = 0.017) with effect size *f* = 0.718, power > 0.99. These values represent the refractive correction used to achieve best-corrected visual acuity. In cases of irregular astigmatism uncorrectable with lenses, a value of 0.00 was assigned, which may underestimate true corneal astigmatism. According to the Amsler–Krumeich classification, the severity distribution in the keratoconus group was as follows: grade 1, 22 eyes; grade 2, four eyes; grade 3, one eye; and grade 4, four eyes.

**Table 1.** Demographic Values of Groups

	C (*n* = 31)	HM (*n* = 26)	KC (*n* = 31)	*P* Value
Age (years)	39.3 ± 7.2	41.5 ± 5.4	38.3 ± 13.0	0.069^*^
Male	19 (61.3%)	18 (69.2%)	19 (61.3%)	0.499^†^
Heart rate (beats/min)	73.1 ± 11.5	73.0 ± 11.9	71.9 ± 11.9	0.809^‡^
Mean blood pressure (mm Hg)	85.11 ± 12.7	85.0 ± 11.8	85.1 ± 11.2	0.073^‡^
Spherical equivalent (D)	−0.87 ± 0.88	−7.16 ± 1.20	−5.67 ± 4.39	<0.001^*^
C–HM				<0.001^§^
C–KC				<0.001^§^
HM–KC				0.112^§^
Refractive cylinder (D)	−0.46 ± 0.45	−0.77 ± 0.52	−2.70 ± 2.36	<0.001^*^
C–HM				0.264^§^
C–KC				<0.001^§^
HM–KC				0.017^§^
logMAR	0.01 ± 0.20	0.13 ± 0.16	0.18 ± 0.35	0.033^*^
C–HM				0.051^§^
C–KC				0.109^§^
HM-KC				1.000^§^
Intraocular pressure (mm Hg)	12.3 ± 2.6	12.1 ± 2.3	10.7 ± 3.7	0.085^*^

C, control; HM, high myopia; KC, keratoconus.

Values are mean ± standard deviation or *n* (%).

* Kruskal-Wallis test.

† χ^2^ test.

‡ ANOVA.

§ Dunn's test.

### MBR

**Figure 2.**
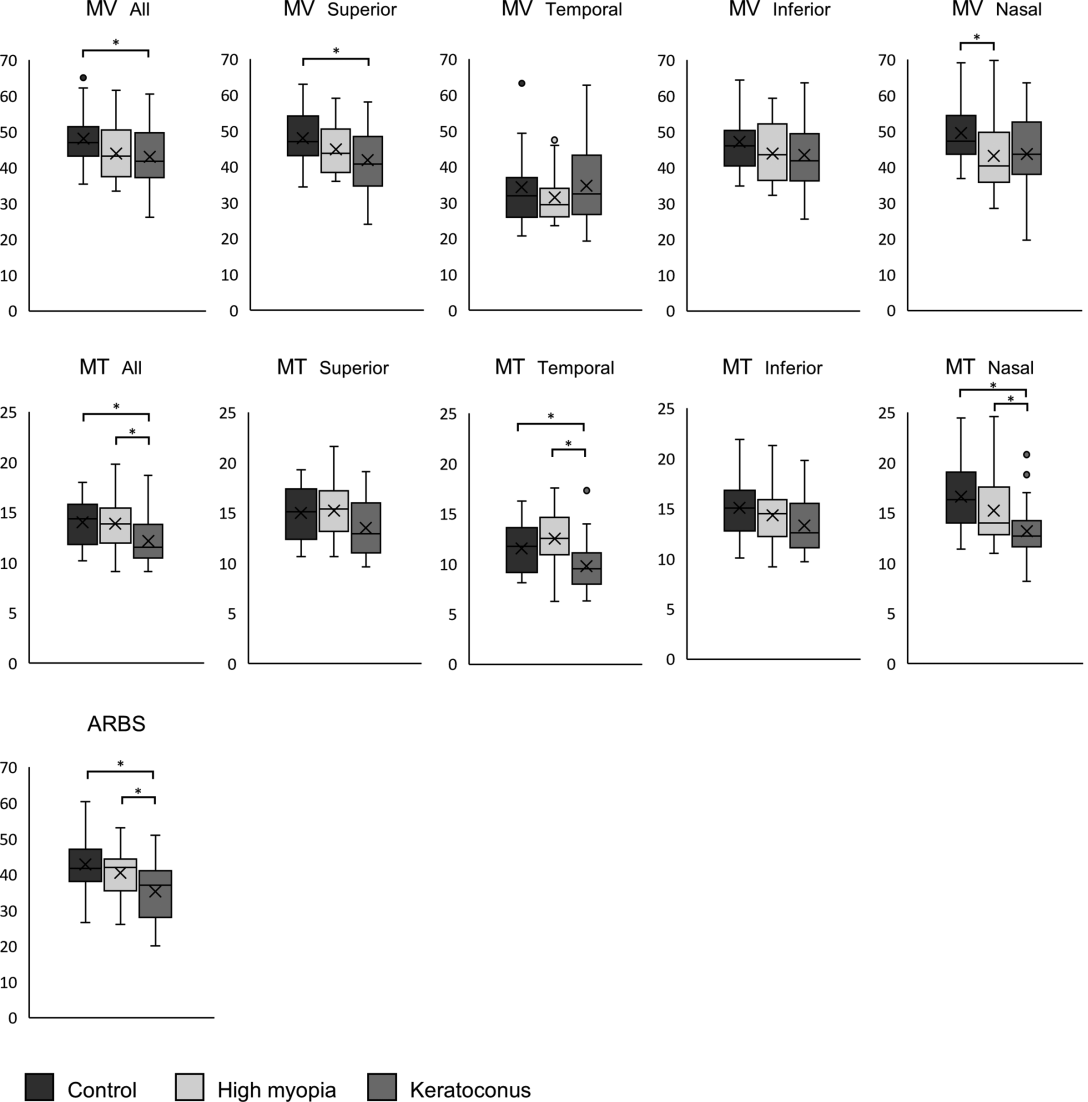
Boxplots of MV, MT, and ARBS values across groups. MBR: For the overall ONH, both MV and MT differed significantly among the three groups (*P* = 0.037 and *P* = 0.005, respectively). MT was significantly lower in the keratoconus group than in both the control and high myopia groups (both *P* = 0.014). MV was significantly lower in the keratoconus group than in controls (*P* = 0.040). In the quadrant analysis, MV differed significantly in the superior and nasal quadrants (*P* = 0.011 and *P* = 0.026). Superior MV was lower in the keratoconus group than in controls (*P* = 0.008), and nasal MV was lower in the high myopia group than in controls (*P* = 0.047). MT differed significantly in the temporal and nasal quadrants (both *P* < 0.001), with lower values in the keratoconus group than in controls (*P* = 0.022, *P* < 0.001) and the high myopia group (*P* < 0.001, *P* < 0.031). ARBS differed significantly among the three groups (*P* = 0.001), with the keratoconus group showing significantly lower values than both the control (*P* = 0.001) and high myopia groups (*P* = 0.036).

For the overall ONH, both MV and MT differed significantly among the three groups (Table 2; Fig. 2; *P* = 0.037 and *P* = 0.005, respectively). Post hoc analysis showed that MT was significantly lower in the keratoconus group than in controls and high myopia (*P* = 0.014, 0.014) with an effect size of *f* = 0.369 and power of 0.87. Although MV also showed a significant difference between the keratoconus group and controls (*P* = 0.040), its statistical power was slightly low (effect size *f* = 0.295, power 0.68).

**Table 2.** MV, MT and ARBS Values of Groups

	C	HM	KC	*P* Value
MV				
All	48.0 ± 7.2	43.9 ± 7.9	43.0 ± 8.9	0.037^*^
C–HM				0.133^†^
C–KC				0.040^†^
HM–KC				0.908^†^
Superior	48.0 ± 7.0	44.8 ± 6.9	41.8 ± 8.8	0.011^*^
C–HM				0.275^†^
C–KC				0.008^†^
HM–KC				0.350^†^
Temporal	34.3 ± 10.8	31.4 ± 6.7	34.7 ± 11.6	0.612^*^
Inferior	47.1 ± 7.8	43.9 ± 8.2	43.5 ± 9.7	0.206^*^
Nasal	49.5 ± 8.7	43.1 ± 10.6	43.6 ± 10.6	0.026^*^
C–HM				0.047^†^
C–KC				0.057^†^
HM–KC				0.982^†^
MT				
All	13.9 ± 2.4	14.0 ± 2.6	12.1 ± 2.3	0.005^*^
C–HM				0.993^†^
C–KC				0.014^†^
HM-KC				0.014^†^
Superior	15.0 ± 2.6	15.2 ± 2.8	13.5 ± 2.9	0.042^*^
C–HM				0.956^†^
C–KC				0.096^†^
HM–KC				0.062^†^
Temporal	11.5 ± 2.4	12.5 ± 2.9	9.8 ± 2.3	<0.001^*^
C–HM				0.325^†^
C–KC				0.022^†^
HM–KC				<0.001^†^
Inferior	15.1 ± 3.0	14.3 ± 2.6	13.3 ± 2.9	0.052^*^
Nasal	16.6 ± 3.1	15.2 ± 3.2	13.2 ± 2.6	<0.001^*^
C–HM				0.190^†^
C–KC				<0.001^†^
HM–KC				0.031^†^
ARBS				
All	42.8 ± 8.0	40.4 ± 6.9	35.3 ± 8.0	0.001^*^
C–HM				0.474^†^
C–KC				0.001^†^
HM–KC				0.036^†^

Values are mean ± standard deviation.

* ANOVA.

† Tukey-Kramer test.

In the quadrant analysis, MV showed significant differences in the superior and nasal quadrants (*P* = 0.011, 0.026). The superior MV was lower in keratoconus than in controls (*P* = 0.008, effect size *f* = 0.369, power 0.87). The nasal MV was lower in the high myopia group than in controls (*P* = 0.047, effect size *f* = 0.295, power 0.68). MT differed significantly in the temporal (effect size *f* = 0.453, power 0.97) and nasal (effect size *f* = 0.500, power > 0.99) quadrants (both *P* < 0.001), with lower values in keratoconus than in controls (*P* = 0.022, <0.001) and the high myopia group (*P* < 0.001, 0.031). Although superior MT also showed significance in ANOVA (*P* = 0.042), the post hoc analysis did not (effect size *f* = 0.274, power 0.61).

### ARBS

ARBS differed significantly among the three groups (*P* = 0.001), with the keratoconus group showing a significantly lower ARBS than both the control (*P* = 0.001) and the high myopia groups (*P* = 0.036), with an effect size of *f* = 0.420 and power = 0.94 (Table 2).

### Pulse Waveform Parameters

No significant differences were found for any parameter (Table 3; see Supplementary Fig. S1).

**Table 3.** Pulse Wave Parameters of Groups

	Control	High Myopia	Keratoconus	*P* Value^*^
Skew				
MV	10.6 ± 1.6	10.4 ± 1.9	11.0 ± 2.6	0.827
MT	11.8 ± 1.6	11.7 ± 1.7	11.2 ± 2.3	0.654
BOS				
MV	82.5 ± 4.1	82.6 ± 4.3	80.9 ± 4.3	0.210
MT	78.1 ± 4.1	79.6 ± 4.1	78.4 ± 4.9	0.443
BOT				
MV	56.1 ± 4.0	55.9 ± 4.8	54.8 ± 6.8	0.838
MT	52.0 ± 3.5	51.5 ± 3.6	52.1 ± 5.0	0.645
RR				
MV	13.5 ± 1.2	13.1 ± 1.5	13.6 ± 2.0	0.504
MT	12.7 ± 0.8	12.8 ± 1.1	12.5 ± 1.4	0.525
FR				
MV	12.4 ± 0.8	12.4 ± 0.9	12.8 ± 1.5	0.543
MT	12.9 ± 0.8	13.0 ± 0.7	12.7 ± 1.2	0.301
FAI				
MV	5.3 ± 1.6	4.7 ± 2.3	5.1 ± 1.7	0.204
MT	1.7 ± 0.4	1.6 ± 0.5	1.5 ± 0.4	0.155
ATI				
MV	29.8 ± 3.6	29.2 ± 4.4	30.1 ± 5.7	0.900
MT	29.5 ± 3.0	30.2 ± 3.9	30.4 ± 6.2	0.884
RI				
MV	0.28 ± 0.06	0.28 ± 0.06	0.30 ± 0.06	0.365
MT	0.34 ± 0.05	0.32 ± 0.06	0.33 ± 0.07	0.431

BOS, blowout score; BOT, blowout time; RR, rising rate; FR, falling rate; FAI, flow acceleration index; ATI, acceleration time index; RI, resistivity index.

Values are mean ± standard deviation.

* Kruskal-Wallis test.

## Discussion

Previous studies reported that keratoconus, although primarily a corneal disease, is also associated with posterior segment changes compared with normal eyes.^3^^–^^8^ However, no study has directly evaluated ONH blood flow in keratoconus. To our knowledge, only two studies assessed ONH vascular density in keratoconus using optical coherence tomography angiography (OCTA).^7^^,^^8^ Both demonstrated that vessel density of the radial peripapillary capillaries was significantly reduced in keratoconus compared with normal eyes. In our study, ARBS was significantly reduced in keratoconus compared with both the control and high myopia groups (Table 2). Because ARBS represents the proportion of retinal vascular area within the ONH, our findings are consistent with the reduced vessel density reported in previous OCTA studies.

Keratoconus is characterized by reduced expression of types I, III, and V collagen, the main components of the corneal stroma.^15^ Elevated MMPs and inflammatory cytokines in the corneal stroma and tear fluid may promote collagen degradation.^16^^,^^17^ Similarly, the vascular wall contains types I, III, IV, and V collagen,^18^ and the extracellular matrix surrounding optic nerve fibers at the ONH contains types I, III, IV, V, and VI collagen.^19^ The lamina cribrosa, which supports retinal ganglion cell axons at the ONH, has a mesh-like collagen structure. In keratoconus, the lamina cribrosa is significantly thinner than in healthy eyes,^6^^,^^20^ suggesting extracellular matrix remodeling at the ONH.

When comparing MV and MT, both of which are derived from MBR, the differences observed in MT were relatively more consistent than those observed in MV. In the overall ONH analysis, MT showed a larger effect size (*f* = 0.369) and higher statistical power (1 − β = 0.87) than MV (*f* = 0.295, power = 0.68), although both parameters reached statistical significance. In the quadrant analysis, MV showed a reduction in the superior quadrant with a moderate effect size and sufficient statistical power (*f* = 0.369, power = 0.87), whereas MT demonstrated larger effect sizes and higher power in the temporal (*f* = 0.453, power = 0.97) and nasal (*f* = 0.500, power > 0.99) quadrants. Taken together, these results indicate that, within MBR-derived parameters, changes in MT were relatively more consistent across regions than those in MV, suggesting that alterations in tissue-level perfusion may be more consistently detected than those in vascular flow. This observation may reflect the involvement of extracellular matrix changes in the vessel wall and lamina cribrosa, which could contribute to the reduction in ONH microcirculation observed in keratoconus.

There is a hypothesis that increased oxidative stress can activate MMPs and may promote extracellular matrix degradation.^21^ Reduced blood flow may exacerbate oxidative stress and could, in turn, contribute to extracellular matrix breakdown. In our cohort, all parameters with significant differences showed consistently lower MBR in keratoconus compared with the control and high myopia groups, indicating a generalized reduction in ONH blood flow.

In contrast, no significant differences were observed in the pulse waveform parameters, despite the significant group differences in MV and MT. Pulse waveform parameters are known to reflect systemic vascular conditions. In this study, heart rate and mean blood pressure did not differ among the three groups (Table 1), which may explain why pulse waveform parameters showed no significant group differences.

Although no direct links have been reported between keratoconus and non-arteritic anterior ischemic optic neuropathy or glaucoma, both of which are characterized by impaired ONH perfusion, multiple studies have reported that all three conditions are frequently associated with obstructive sleep apnea (OSA).^22^^–^^31^ Elevated serum levels of MMPs, which are implicated in the pathogenesis of keratoconus, have also been reported in patients with OSA,^21^ suggesting a potential overlap in pathogenic mechanisms. OSA has been associated with an increased risk of non-arteritic anterior ischemic optic neuropathy,^25^^–^^27^ with proposed mechanisms including recurrent nocturnal hypoxia, impaired vascular autoregulation, and fluctuations in intracranial pressure during apneic episodes that may affect ONH perfusion.^25^ Associations with glaucoma have likewise been reported,^28^^,^^29^ with possible contributions from elevated episcleral venous pressure and hypoxia-related vascular stress.^30^^,^^31^ Mechanical pressure on the eye during sleep in keratoconus has also been proposed as a contributing factor to disease development,^32^ and such ocular compression is associated with elevations in intraocular pressure. However, because such factors were not directly evaluated in the present study, these potential associations remain speculative, and future investigations are needed to directly assess them.

Anterior segment OCT studies have reported increased scleral thickness in patients with severe OSA.^33^ Although this finding represents a structural change opposite to the corneal thinning observed in keratoconus, both may reflect dysregulated extracellular matrix turnover under systemic or metabolic stress. Floppy eyelid syndrome, which has been frequently associated with both keratoconus and OSA, is characterized by eyelid laxity and elastin fiber degeneration.^24^ Together, these observations support the concept that collagen- and elastin-rich tissues, including the cornea, sclera, lamina cribrosa, and eyelid, may constitute a continuous structural and biomechanical network that warrants further investigation in relation to ONH microcirculation.

One strength of this study is that we demonstrated not only reduced ARBS but also decreased microcirculation in the ONH of keratoconus eyes. However, this study has a few limitations. First, obtaining high-quality LSFG images was difficult in eyes with advanced corneal deformation due to the technical limitations of the device. Because cases with poor image quality were excluded, advanced keratoconus was not analyzed. Second, eyes with keratoconus cannot be completely excluded from the control and the high myopia groups. However, we believe the prevalence is extremely low and can be disregarded in statistical analyses.^2^ Third, the sample size was relatively small. Further studies with large cohorts and longitudinal designs are warranted to elucidate the relationship between keratoconus and ocular circulation changes.

## Conclusions

This study showed that both MBR and ARBS in the ONH were significantly reduced in keratoconus compared with control and high myopia eyes, suggesting retinal blood flow reduction in keratoconus. Clinicians should pay attention not only to corneal morphology but also to ONH circulation when managing patients with keratoconus.

## Supplementary Material

Supplement 1
