# Supplementary figures and images for: Evaluation of Microcirculation at the Optic Nerve Head in Eyes With Keratoconus
Source: Invest Ophthalmol Vis Sci. 2026 Apr 13;67(4):25. doi: 10.1167/iovs.67.4.25 (PMC13101836; doi:10.1167/iovs.67.4.25)

**Supplementary Material**
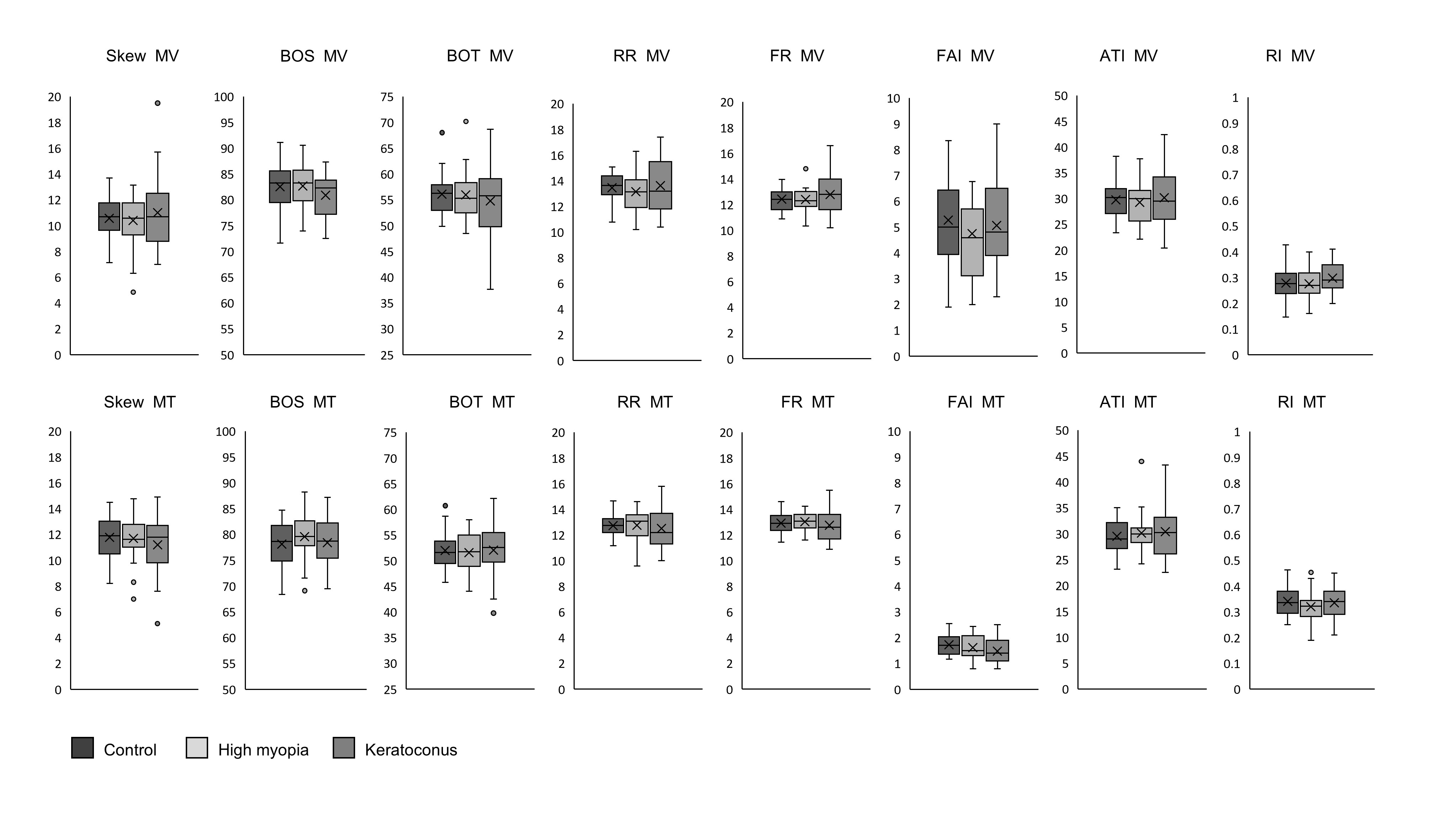


**Figure S1.** Boxplots of pulse waveform parameters across groups.

Supplement: Supplement 1 [file iovs-67-4-25_s001.docx]
